# Aging and Calorie Restriction Oppositely Affect Mitochondrial Biogenesis through TFAM Binding at Both Origins of Mitochondrial DNA Replication in Rat Liver

**DOI:** 10.1371/journal.pone.0074644

**Published:** 2013-09-13

**Authors:** Anna Picca, Vito Pesce, Flavio Fracasso, Anna-Maria Joseph, Christiaan Leeuwenburgh, Angela M. S. Lezza

**Affiliations:** 1 Department of Aging and Geriatric Research, Institute on Aging, Division of Biology of Aging, University of Florida, Gainesville, United States of America; 2 Department of Biosciences, Biotechnologies and Biopharmaceutics, University of Bari, Bari, Italy; University of Medicine and Dentistry of New Jersey, United States of America

## Abstract

Aging affects mitochondria in a tissue-specific manner. Calorie restriction (CR) is, so far, the only intervention able to delay or prevent the onset of several age-related changes also in mitochondria. Using livers from middle age (18-month-old), 28-month-old and 32-month-old *ad libitum*-fed and 28-month-old calorie-restricted rats we found an age-related decrease in mitochondrial DNA (mtDNA) content and mitochondrial transcription factor A (TFAM) amount, fully prevented by CR. We revealed also an age-related decrease, completely prevented by CR, for the proteins PGC-1α NRF-1 and cytochrome c oxidase subunit IV, supporting the efficiency of CR to forestall the age-related decrease in mitochondrial biogenesis. Furthermore, CR counteracted the age-related increase in oxidative damage to proteins, represented by the increased amount of oxidized peroxiredoxins (PRX-SO_3_) in the *ad libitum*-fed animals. An unexpected age-related decrease in the mitochondrial proteins peroxiredoxin III (Prx III) and superoxide dismutase 2 (SOD2), usually induced by increased ROS and involved in mitochondrial biogenesis, suggested a prevailing relevance of the age-reduced mitochondrial biogenesis above the induction by ROS in the regulation of expression of these genes with aging. The partial prevention of the decrease in Prx III and SOD2 proteins by CR also supported the preservation of mitochondrial biogenesis in the anti-aging action of CR. To investigate further the age- and CR-related effects on mitochondrial biogenesis we analyzed the *in vivo* binding of TFAM to specific mtDNA regions and demonstrated a marked increase in the TFAM-bound amounts of mtDNA at both origins of replication with aging, fully prevented by CR. A novel, positive correlation between the paired amounts of TFAM-bound mtDNA at these sub-regions was found in the joined middle age *ad libitum*-fed and 28-month-old calorie-restricted groups, but not in the 28-month-old *ad libitum*-fed counterpart suggesting a quite different modulation of TFAM binding at both origins of replication in aging and CR.

## Introduction

Aging involves the progressive functional decline of tissues, which includes the dysfunction of the mitochondrial respiratory complexes leading to a reduced ATP synthesis. This age-related feature severely affects those tissues as brain, heart and skeletal muscle, highly dependent on oxidative metabolism for their energy supply [1-3]. Liver also demonstrates a marked age-related decrease in mitochondrial function [4], together with another feature of the “mitochondrial theory of aging” [5-7]: the increased presence of reactive oxygen species (ROS), by-products of the mitochondrial respiratory complexes. The induced oxidative stress results in oxidative damage to mitochondrial DNA (mtDNA), proteins and lipids and contributes to the aging phenotype. Among the ROS-related damages to mtDNA can be included various qualitative alterations (bases modifications, abasic sites, single- and double-strand breaks, point mutations and large size deletions, reviewed in 8). Quantitative changes in the relative content of mtDNA with aging have been found in various tissues from humans and animals [9-13] and, in particular, rat liver mtDNA has been shown to decrease with age [10,14,15]. Recent studies have proposed a wider model of aging where the mechanism of increased DNA damage, associated to nuclear telomeres shortening, intersects with that of the altered metabolic pathways at mitochondrial level. According to such integrated view, the response to the increased DNA damage should induce activation of p53 leading to decreased mitochondrial biogenesis through suppression of the peroxisome proliferator-activated receptor-γ co-activator-1α (PPAR-γ co-activator-1α PGC-1α), which is a central regulator of mitochondrial biogenesis and function [16]. This transcriptional co-activator presents a very complex and multiform regulation of expression at different levels, thus enabling graduated responses to a variety of metabolic signals, including ROS amount and nutrients availability. Effectively, the expression of PGC-1α, sensitive to multiple stimuli, provides the main communication route for adaptations of the organelle metabolism and/or biogenesis. Among the nuclear factors involved in the PGC-1α-dependent cascade of mitochondrial biogenesis there is the High Mobility Group protein mitochondrial transcription factor A (TFAM) [17,18]. TFAM was initially characterized for its function in mitochondrial transcription [19] and later identified as a main player in the regulation of mtDNA copy number [20] because of the close connection between transcription and replication of mtDNA [21]. Furthermore, TFAM is deeply involved in the composition of mtDNA nucleoids [22] and likely in the sensing and repair of oxidative damage to mtDNA [23,24]. TFAM protein expression has been investigated in some tissues of aged rats [25] like different hind limb muscles [26] and, very recently, liver [15] and brain frontal cortex [13] further confirming the tissue-specificity of aging. To date, the only established experimental approach efficacious in delaying or preventing the onset of several age-related alterations of mitochondria in organisms, ranging from yeast to man, is calorie restriction (CR) [27-29]. The reduction in the age-related oxidative stress is largely indicated at the basis of the efficacy of CR [29,30] and can be related to the effects of CR on mitochondrial age-related phenotypic and genotypic alterations [31-37]. In particular, the tissue-specific effects of CR include the prevention of the age-related loss of mtDNA in rat liver [14] and the partial preservation of TFAM binding to mtDNA in rat brain with its relevant consequences for mitochondrial biogenesis [13]. Therefore, to study the eventual changes of mtDNA content and of TFAM and other proteins amounts as well as of TFAM binding to mtDNA with aging and CR in liver was very intriguing for us, also to get a deeper insight into the protective effects of the calorie restriction itself.

## Materials and Methods

### Samples

The study was approved by the Institutional Animal Care and Use Committee at the University of Florida. All procedures were performed in accordance with the National Institutes of Health guidelines for the care and use of laboratory animals. Twenty-four Fischer 344 x Brown Norway (F344BNF1) male rats were obtained from the National Institute of Aging colony (Indianapolis, IN) and acclimated to the animal housing room of the University of Florida for 2 weeks. The rats were housed individually in a temperature- (20 +/- 2°C) and light-controlled environment (12-hour light/dark cycle) with regular rat chow and water available *ad libitum*. The animals consisted of the following groups: middle age that is *ad libitum*-fed 18-month-old (AL-MA, n = 6), old that is *ad libitum*-fed 28-month-old (AL-28, n = 8), very old that is *ad libitum*-fed 32-month-old (AL-32, n = 3) and old that is calorie-restricted 28-month-old (CRO, n = 7) rats. Calorie restriction had been initiated at 3.5 months of age (10% restriction), raised to 25% restriction at 3.75 months and kept at 40% restriction from 4 months until the end of each animal’s life that is at 28 months of age (CRO). Animals were anesthetized before being sacrificed and samples from the liver immediately removed, snap-frozen in isopentane cooled by liquid nitrogen and stored in liquid nitrogen until further use.

### Determination of mtDNA content

MtDNA content was measured following the procedure described by Picca et al. [13],, using quantitative Real Time PCR. Real Time PCR amplification reactions were performed via SYBR Green chemistry on an ABI PRISM 7300 Sequence Detection System (Applied Biosystems, Foster City, CA, USA). The primers were specific, respectively, for the rat mitochondrial D-loop region (numbering is according to GenBank^TM^ accession number AY172581) and for the rat nuclear β-actin gene (numbering is according to GenBank^TM^ accession number VO1217.1) and are listed in Table 1. Each sample was analyzed in triplicate in 25 µl of final volume containing: iTaq SYBR Green Supermix PCR 1X Master Mix (Bio-Rad Laboratories Inc., Hercules, CA, USA), 0.2 µM forward and reverse primers and DNA template (2.5 µl of diluted 1:50). After 10 min of denaturation at 95°C, amplification proceeded for 40 cycles, each consisting of denaturation at 95°C for 15 s, annealing and extension at 60°C for 1 min. The quantification of the relative mtDNA content in AL-28, AL-32 and CRO rats, compared to AL-MA rats, all normalized to β-actin, was performed according to the Pfaffl mathematical model [38].

**Table 1:** 

Primers for PCR of mIP								
Primer set	Forward primer		Reverse primer		(nps)		(nps)	
8.0 For/8.2 Rev	5’- CAACCGACTACACTCATTTCAAC-3’		5’- CTCATAGGGGGATGGCTATGC-3’		(8035-8057)		(8246-8226)	
16.0 For/16.2 Rev	5’- GCTCGAAAGACTATTTTATTCATG-3’		5’- GCTAAGATTTAAGTTAAAATTTTGTG-3’		(16015-16038)		(16226-16201)	
5.0 For/5.3Rev	5’- GGATTCAAACCTACGAAAATTTAG-3’		5’- GTGGTTAGTTGAAAAGAGTCAAC-3’		(5092-5115)		(5358-5336)	
Primers for RT-PCR								
mtDNA For/mtDNA Rev		5’- GGTTCTTACTTCAGGGCCATCA-3’		5’ – TGATTAGACCCGTTACCATCGA- 3’		(15785-15806)		(15868-15847)
Beta-actin For/Beta-actin Rev		5’ – CCCAGCCATGTACGTAGCCA -3’		5’ – CGTCTCCGGAGTCCATCAC -3’		(2181-2200)		(2266-2248)
RT-Dloop For/RT-Dloop Rev		5’- CACCCCCTACACCTGAAACTT-3’		5’- TTTGTGTCGGGAAATTTTACCAAT-3’		(16092-16112)		(16250-16227)
RT-OriL For/RT-OriL Rev		5’- CAGCTAAATACCCTACTTACTGG-3’		5’- GCCCCCTTTTTACCAAAAAGCC-3’		(5120-5142)		(5270-5249)

### Western blotting

Total proteins were extracted from liver samples obtained from AL-MA, AL-28, AL-32 and CRO animals. Approximately 100 mg of each frozen sample were grounded with a mortar and pestle and suspended in 600 µl of lysis buffer (220 mM mannitol, 70 mM sucrose, 20 mM Tris–HCl pH 7.4, 5 mM MgCl_2_, 5 mM EGTA and 1 mM EDTA). Cell lysates were pre-cleared by centrifugation in an Eppendorf microfuge at 12,000 rpm for 10 min and the supernatant fraction containing proteins was recovered. Proteins were quantified with the Bradford method (Bio-Rad Laboratories Inc., Hercules, CA, USA) according to the supplier’s instructions. Total proteins (15 µg) were separated by gel electrophoresis on 4-12% Bis-Tris Criterion XT precast gels (Bio-Rad Laboratories Inc., Hercules, CA, USA) at 150 V for 1.0 h in 1x XT-MOPS and electroblotted onto PVDF membranes (Amersham-Pharmacia Biotech Inc., Piscataway, NJ, USA). Primary antibodies were, respectively, from Santa Cruz Biotechnology Inc., (Santa Cruz, CA, USA) (NRF-1, PGC-1α, COX IV), from Ab Frontiers, (Seoul, Korea) (Prx III and PRX-SO_3_), from Sigma-Aldrich Corp., (St. Louis, MO, USA) (β-actin), from Assay Designs, (Ann Arbor, MI, USA) (SOD2) and from Abcam Inc. (Cambridge, MA, USA) (VDAC-Porin). The antibody against TFAM was custom-made using as antigen in rabbit the protein expressed from the clone containing the peptide fraction corresponding to aa 35 to 201 of the rat protein and donated by Dr. H. Hinagaki (Department of Chemistry, National Industrial Research Institute of Nagoya, Japan). The membranes were blocked for 1 hr in 5% milk in 1X-PBS/Tween 20 (0.15 M NaCl, 0.1 mM KH_2_PO_4_, 3 mM Na_2_HPO_4_, 0.1% Tween 20) and probed with TFAM (1:50,000), PGC-1α (1:2,000), NRF-1(1:50,000), COX IV (1:5,000), Prx III (1:100,000), PRX-SO_3_ (1:100,000), SOD2 (1:100,000), VDAC (1:50,000) and β-actin (1:50,000). Membranes were then incubated with anti-rabbit secondary antibody (for TFAM, NRF-1, Prx III, PRX-SO_3,_ SOD2, VDAC and β-actin) at a dilution of 1:10,000 either anti-goat secondary antibody (for PGC-1α and COX IV) at a dilution of 1:5,000 conjugated with horseradish peroxidase (HRP) (Santa Cruz Biotechnology, Inc., Santa Cruz, CA, USA). Membranes were washed in PBS (three times X 15 min/each) and proteins subsequently visualized with an enhanced chemiluminescence kit (ECL-Plus; Amersham-Pharmacia Biotech Inc., Piscataway, NJ, USA). Autoradiographs were analyzed by laser densitometry with the Chemi Doc System and Quantity One software (Bio-Rad Laboratories Inc., Hercules, CA, USA). The densitometric value of OD units of every protein band was then related to the OD units number of the respective band of the β-actin for each analyzed sample. Densitometry was performed on every triplicate gel and the mean value was shown in the corresponding histogram.

### Mitochondrial DNA Immunoprecipitation (mIP)

The binding of TFAM to specific regions of mtDNA was analyzed using mitochondrial DNA immunoprecipitation (mIP) following the procedure described by Picca et al. [13]. Frozen samples of 100 mg each were subjected, successively, to: cross-linking, termination of cross-linking, brief homogenization and centrifugation. Each resulting pellet was then washed with PBS, suspended with homogenization buffer and manually homogenized. The homogenized sample was briefly centrifuged and the pellet was incubated in lysis buffer. Cellular DNA was sheared by sonication and the size range of produced DNA fragments (between 500 and 900 base pairs) was checked by electrophoresis on a 1.2% agarose gel in 1X TAE buffer. Each sample was diluted with 3 volumes of FSB buffer (5 mM EDTA, 20 mM Tris HCl pH 7.5, 50 mM NaCl), pre-cleared with 75 µl of protein A-agarose/Salmon sperm 50% DNA (Upstate, distributed by Millipore Corporate Headquarters, Billerica, MA, USA) for 2 ml of sample on a rotator at 4°C for 30 minutes and centrifuged at 1,000 rpm for 1 minute. Each centrifuged sample was resuspended with 400 µl of FSB and divided into four aliquots: the input (100 µl) was not immunoprecipitated, but stored at -80 °C until the cross-linking reversal; the other three aliquots (100 µl each) were incubated overnight at 4°C, respectively, with a rabbit anti-TFAM antibody (1:50 dilution), a non-specific rabbit anti-β-actin antibody (1:100 dilution) and without antibody (-Ab). The next day 15 µl of Protein A-agarose were added to each sample for 2 h at 4°C to isolate protein-DNA complexes. The samples were briefly centrifuged and the pellets were washed: three times with 1 ml of RIPA buffer (140 mM NaCl), three times with 1 ml of RIPA buffer (500 mM NaCl), three times with 1 ml of LiCl buffer and twice with 1 ml of TE. The last pellets were suspended with TE containing 0.5% SDS (200 µl) and, together with the original input, incubated at 65°C for 6 hours for the thermal reversal of the cross-links. The DNA samples treated with specific antibodies, the -Ab samples and the input DNAs were all ethanol-precipitated overnight. The resulting pellets were washed with cold 70% ethanol, dried and suspended in 200 µl of sterile ultrapure H_2_O to be treated with 10 µg of RNase A (50 µg/µl) for one hour at 37°C and to be incubated with 20 µg of Proteinase K (100 µg/µl) and SDS (0.25%) at 37°C overnight. A first extraction with phenol/chloroform/isoamyl alcohol (25:24:1) and a second extraction with chloroform/isoamyl alcohol (24:1) were performed. All DNAs were ethanol-precipitated overnight, centrifuged, washed with cold 70% ethanol, dried and suspended in 60 µl of 10 mM Tris-HCl pH 8.0. Input and mIP mtDNAs were subjected to PCR analysis using three primers pairs. Table 1 shows the primers sequences. PCR reactions were performed in a 25 µl mixture containing: 1 µl DNA template, 20 pmol of each primer, 200 µM dNTPs, 1.5 mM MgCl_2_, 1X Taq Buffer and 1U Taq Polymerase (Roche, F. Hoffmann-La Roche, Basel, Switzerland), using a mastercycler PCR (Eppendorf Scientific, Hinz GmbH, Hamburg, Germany). Amplification conditions were: one cycle at 94°C for 5 min followed by 30 cycles at 94°C for 1 min, 60°C for 1 min, 72°C for 1 min, and then finally one cycle at 72°C for 5 min. Reactions were analyzed, by densitometry, on an ethidium bromide-stained 1.3% agarose gel in 1X TAE buffer for the detection of specific TFAM binding, by subtracting the value of the intensity of the aliquot precipitated without primary antibody from that of the TFAM-immunoprecipitated aliquot, both normalized to the value of the respective input aliquot made equal to 1. Such value was calculated for each sample from the three experimental replicas and the respective mean was used in the box-plot representation of the corresponding group of rats.

### Quantitative Real Time PCR of mIP DNA

The relative amounts of mtDNA immunoprecipitated by TFAM were determined by quantitative Real Time PCR (qRT-PCR) using two pairs of specific primers for the D-loop and OriL sub-regions (Table 1), accurately designed with the Primer Express software (Applied Biosystems, Foster City, CA, USA). The qRT-PCR amplification reactions were performed via SYBR Green chemistry on an ABI PRISM 7000 Sequence Detection System (Applied Biosystems, Foster City, CA, USA). The method was validated by primer limiting experiments and by evaluating the equal reaction efficiency of the amplicons. Amplification specificity was accurately controlled. Each sample was analyzed in triplicate in 25 µl of final volume containing: iTaq SYBR Green Supermix PCR 1X Master Mix (Bio-Rad Laboratories Inc., Hercules, CA, USA), 0.2 µM forward and reverse primers and 2.5 µl of DNA template from the input or immunoprecipitated with anti-TFAM or without antibodies (diluted 1:10) DNA aliquots. After a 10 min denaturation at 95°C, samples were amplified for 40 cycles, each consisting of denaturation at 95°C for 15 s, annealing and extension at 60°C for 1 min. The specific amounts of TFAM-bound mtDNA were calculated according to Vercauteren et al. [39] and to Picca et al. [13]. Briefly, the calculation of each relative amount of TFAM-bound mtDNA was performed according to the formula 2^ΔCTx^ -2^ΔCTb^, where ΔCTx is the C_T_ difference between the C_T_ values of the input and of the immunoprecipitated sample and ΔCTb is the C_T_ difference between the C_T_ values of the input and of the -Ab sample.

### Statistics

The statistical significance of differences between groups of animals was assessed by analysis of variance (ANOVA) using Tukey Honestly Significant Difference (HSD) *post-hoc* test with the SPSS Base 11.5 software (SPSS Inc., Chicago, IL, USA). Correlations between variables were analyzed using Pearson’s test. Statistical significance for all tests was set at *p*<0.05.

## Results

Relative mtDNA content and TFAM amount in liver from aged *ad libitum*-fed and CR-treated rats.

**Figure 1:**
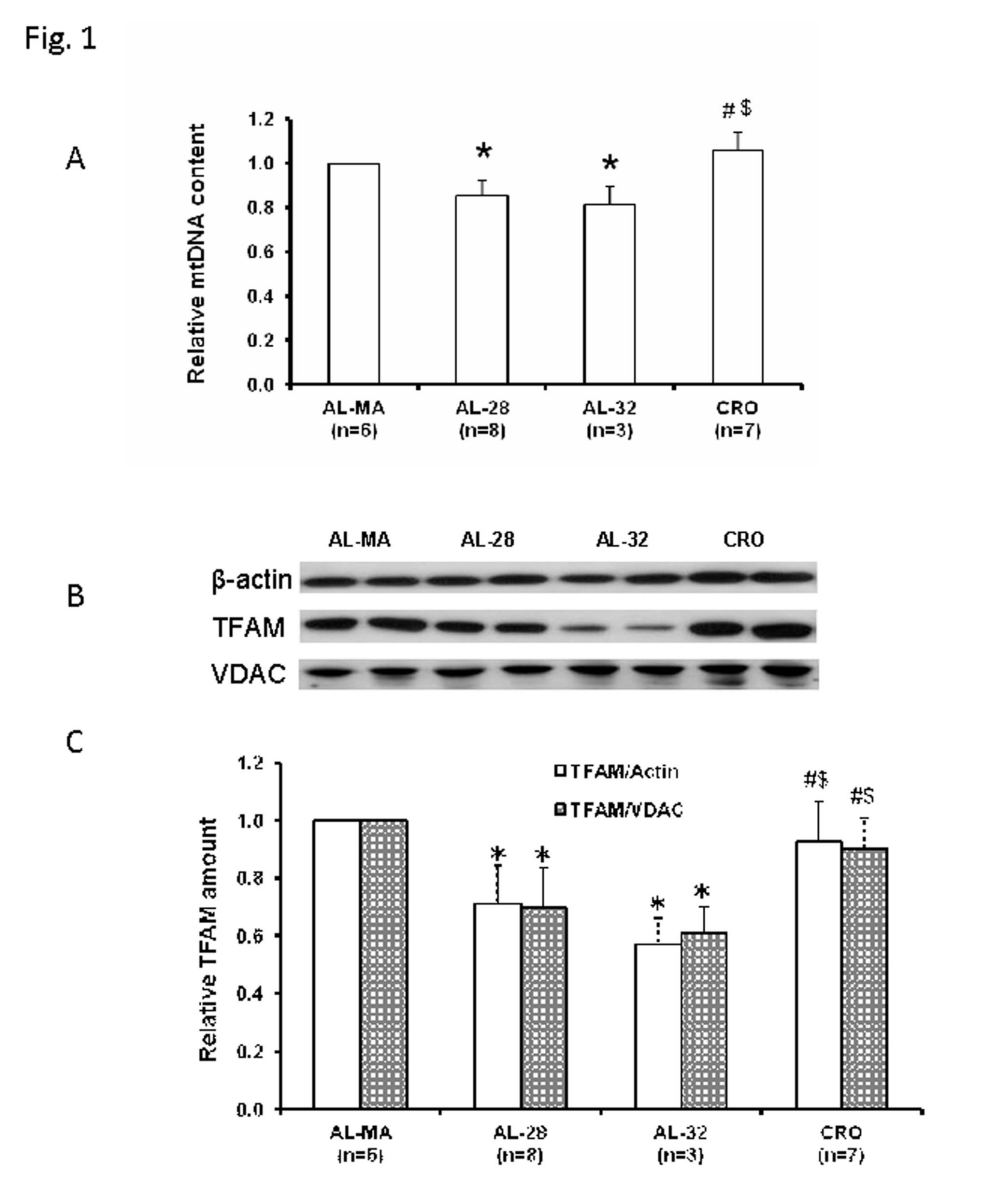
Age- and calorie restriction-related changes of mtDNA content and TFAM amount in rat liver. Aging induced a decrease in the mtDNA content of *ad* *libitum*-fed rats, fully prevented by CR. Also the TFAM amount presented an age-related decrease in the *ad* *libitum*-fed animals, completely prevented by CR. **Α** The histogram shows the mean value of the ratio mtDNA/nuclear DNA, determined by qRT-PCR, in AL-28, AL-32 and CRO rats, compared to AL-MA rats. Bars represent the mean (+/- SD) obtained, respectively, from analysis in triplicate of total nucleic acids from each AL-MA, AL-28, AL-32 and CRO rat. *p<0.05 versus the value of the AL-MA rats (fixed as 1), # *p*<0.05 versus the value of the AL-28 rats, § *p*<0.05 versus the value of the AL-32 rats; n=number of analyzed animals. **B** Representative western blot carried out in two rats from each assayed group. The bands from top to bottom show, respectively, the signals from β-actin, TFAM and VDAC. **C** The histogram shows the relative amount of TFAM in AL-28, AL-32 and CRO rats, determined by densitometry analysis of the results from triplicated western blots experiments, compared to AL-MA rats. The densitometric value of OD units of every TFAM band was related to the number of OD units of the respective band of the β-actin as well as to that of the respective band of VDAC for each analyzed sample. Bars represent the mean (+/- SD) of the relative (TFAM/β-actin and TFAM/VDAC) values obtained from each AL-MA, AL-28, AL-32 and CRO rat. Comparisons were made with respect to the value of the AL-MA rats (fixed as 1). *p<0.05 versus the value of the AL-MA rats, # *p*<0.05 versus the value of the AL-28 rats, § *p*<0.05 versus the value of the AL-32 rats; n=number of analyzed animals.

Aging influences the mtDNA content in several tissues from various organisms [10-12,15] and CR has been shown also to affect the mtDNA content [13,14]. Therefore we decided to determine, by Real Time-PCR [13], the relative mtDNA content in the liver samples from all four groups of animals. In Figure 1 A it is evident a 15% statistically significant decrease in the mtDNA content of old *ad libitum*-fed rats (AL-28) and a 19%-reduced value in the very old group of *ad libitum*-fed animals (AL-32), with respects to the controls namely the middle age *ad libitum*-fed (AL-MA) rats. Such age-related decrease was fully prevented by the CR as the mean mtDNA content in the old calorie-restricted rats (CRO) was statistically not different from the AL-MA value, but significantly higher than that of the *ad libitum*-fed age-matched counterparts (21% higher content with respects to the AL-28 animals value) and even higher than that of the AL-32 rats (25% increased content). In consideration of the close connection between mtDNA and TFAM contents, we determined, by western blot experiments, the amount of TFAM in the livers of all groups (Figure 1 B,C) using the β-actin unchanged amount as the internal standard reference for each sample. In the same experiments we assayed the mitochondrial content of all samples by testing the protein of the outer mitochondrial membrane VDAC and thus verified the absence of any age- either CR-related change (Figure 1 B,C). Since both β-actin and VDAC amounts did not change in aging either CR we choose β-actin as the normalizing protein in all upcoming western blots experiments. Therefore, the densitometric value of OD units of TFAM band and of every other assayed protein band was related to the number of OD units of the respective band of the β-actin for each analyzed sample. An age-related, statistically significant decrease in the TFAM amount was found, progressively presenting a 29%-decreased value in the AL-28 group and a 43%-decreased value in the AL-32 animals. Such decrease was completely prevented by CR since the amount of TFAM in the CRO rats was not statistically different from that of the AL-MA animals and significantly higher than those of the AL-28 as well as of the AL-32 rats, further supporting the preventive effect of the CR.

### Aging and CR effects on the expression of mitochondrial biogenesis and ROS-sensitive proteins

**Figure 2:**
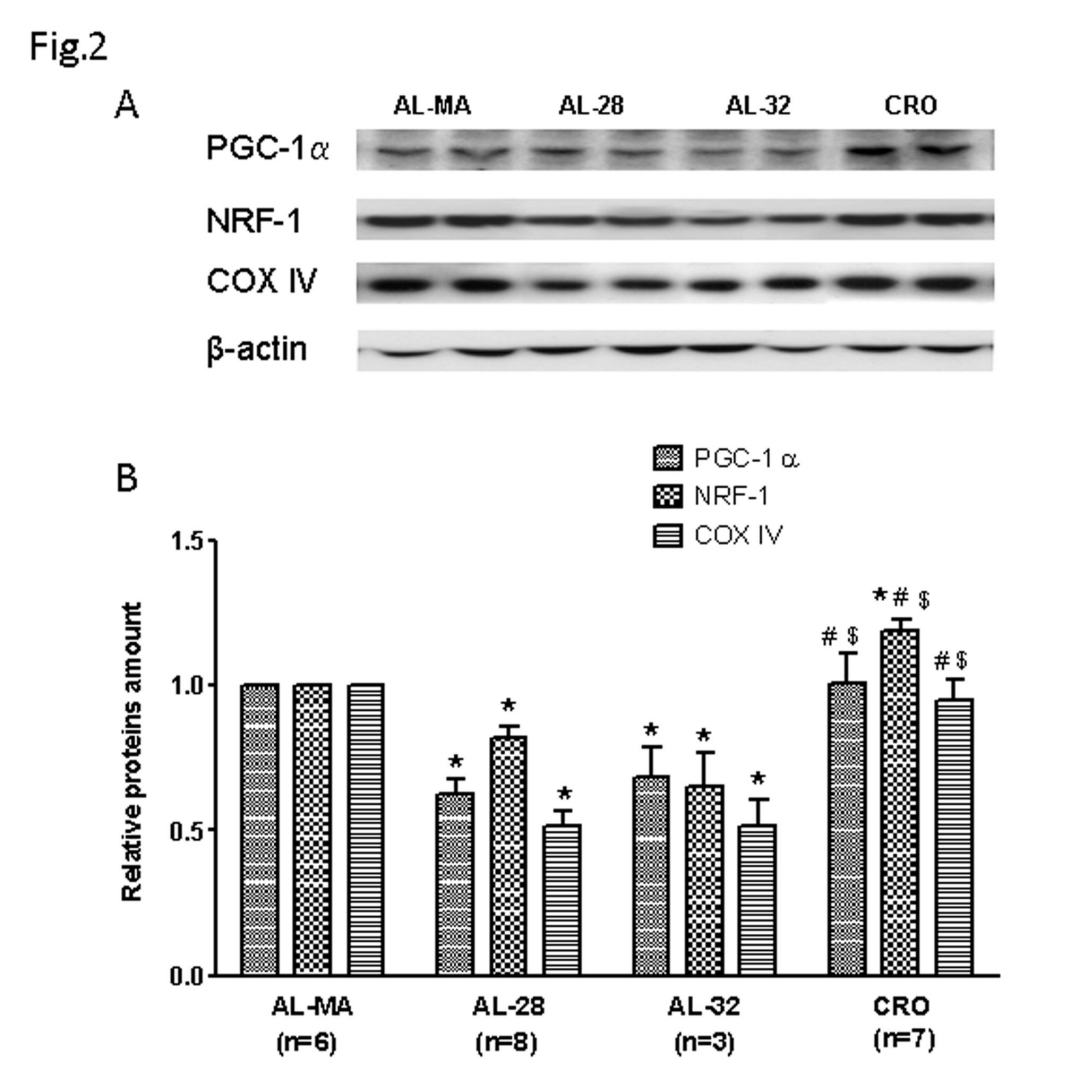
Age- and calorie restriction-related changes of PGC-1α NRF-1 and COX IV amounts in rat liver. Aging induced a decreased expression, completely prevented by CR, of the analyzed proteins, involved in mitochondrial biogenesis. **A** Representative western blot carried out in two rats from each assayed group. The bands from top to bottom show, respectively, the signals from PGC-1α NRF-1, COX IV and β-actin. **B** The histogram shows the relative amounts of PGC-1α, NRF-1 and COX IV in AL-28, AL-32 and CRO rats, compared to AL-MA rats, all normalized with respect to β-actin. Bars represent the mean (+/- SD) of the values obtained, respectively, from analysis in triplicate of the protein extract, from each AL-MA, AL-28, AL-32 and CRO rat. *p<0.05 versus the value of the AL-MA rats (fixed as 1), # *p*<0.05 versus the value of the AL-28 rats, $ *p*<0.05 versus the value of the AL-32 rats; n=number of analyzed animals.

**Figure 3:**
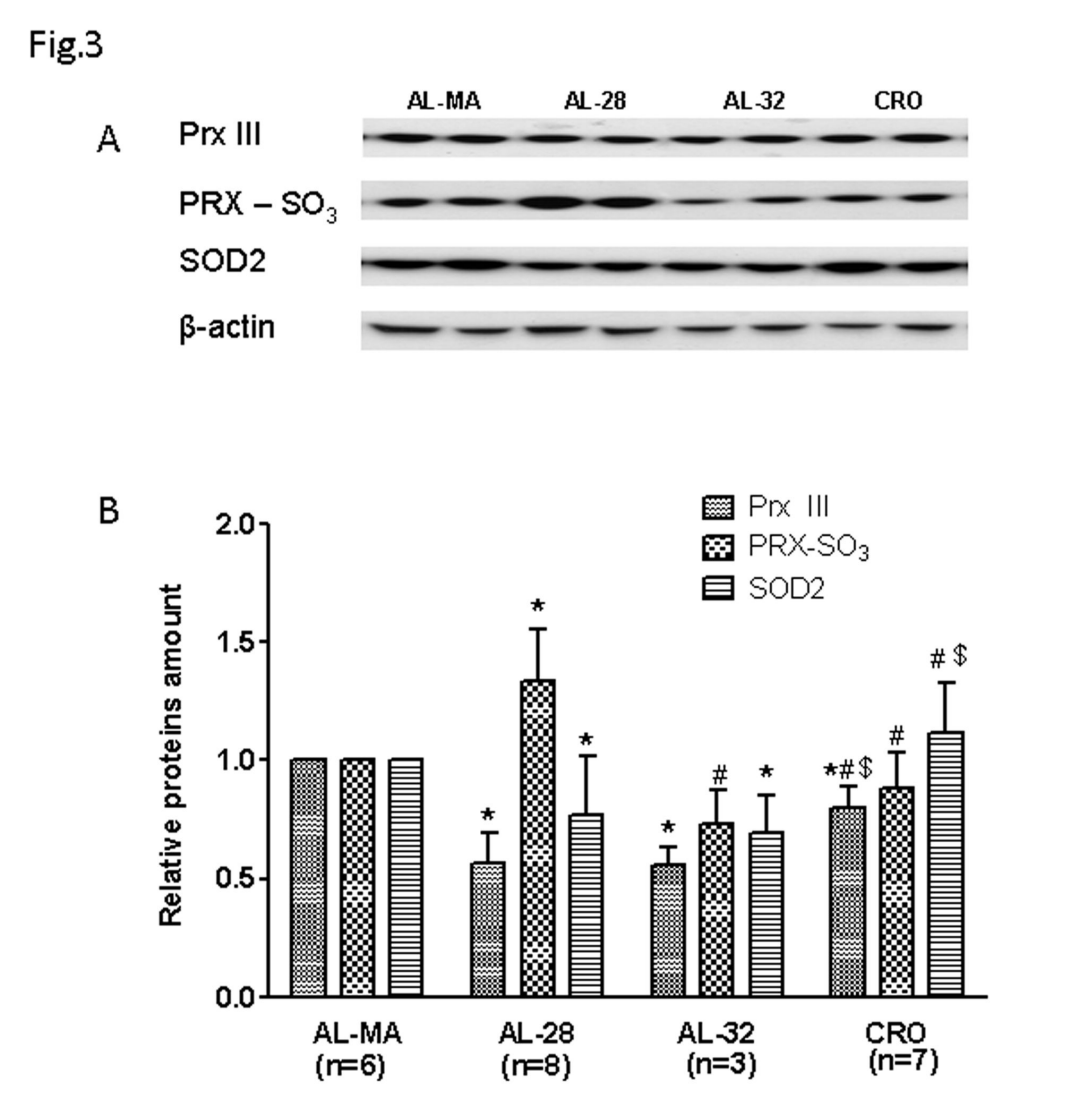
Age- and calorie restriction-related changes of Prx III PRX-SO_3_ and SOD2 amounts in rat liver. Prx III expression presented an age-related decrease, attenuated by CR. An age-related increase in PRX-SO_3_ was found only in the AL-28 rats and prevented by CR. Aging induced a decrease in SOD2 expression, completely prevented by CR. **A** Representative western blot carried out in two rats from each assayed group. The bands from top to bottom show, respectively, the signals from Prx III PRX-SO_3_, SOD2 and β-actin. **B** The histogram shows the relative amounts of Prx III PRX-SO_3_ and SOD2 in AL-28, AL-32 and CRO rats, compared to AL-MA rats, all normalized with respect to β-actin. Bars represent the mean (+/- SD) of the values obtained, respectively, from analysis in triplicate of the protein extract, from each AL-MA, AL-28, AL-32 and CRO rat. *p<0.05 versus the value of the AL-MA rats (fixed as 1), # *p*<0.05 versus the value of the AL-28 rats, $ *p*<0.05 versus the value of the AL-32 rats; n=number of analyzed animals.

Since TFAM is the final step in the PGC-1α-dependent cascade leading to mitochondrial biogenesis, we then evaluated the protein expression of the factors NRF-1 and PGC-1α as well as of the COX IV subunit from the cytochrome c oxidase complex. This subunit is coded for by nuclear DNA, usually expressed in a coordinated way with the mtDNA-encoded subunits of the same respiratory complex and directly regulated by PGC-1α [40] and NRF-1 [41]. As for PGC-1α the master regulator of mitochondrial biogenesis we showed an age-related decrease of expression (Figure 2 A,B), presenting a statistically significant 37%-reduced value in the AL-28 rats and a similar significant 32%-reduced value in the AL-32 ones. Such decrease was completely prevented by CR as the CRO rats had a PGC-1α content not significantly different from that of the AL-MA animals, but significantly higher than those of both the AL-28 and the AL-32 animals. Also for the NRF-1 factor, an age-related decrease in its expression was demonstrated (Figure 2 A,B) finding a statistically significant 18%-reduced value in the AL-28 rats and a statistically significant 35%-reduced one in the AL-32 animals. Such reduction was fully prevented by CR since the mean NRF-1 amount in the CRO rats was significantly higher than all the AL-MA, AL-28 and AL-32 contents. We measured the amount of the COX IV protein (Figure 2 A,B), finding a relevant age-related decrease in its expression that is a statistically significant 49%-reduced amount, shared by both AL-28 and AL-32 groups, completely prevented by the CR since the CRO animals presented a value not statistically different from that of the AL-MA group, but significantly higher than those of both AL-28 and AL-32 rats. As aging is characterized by a chronic oxidative stress, we determined the amount of a very efficient mitochondrial ROS-scavenging protein, Peroxiredoxin III (Prx III) [42], in all samples from the four groups. The results are presented in Figure 3 (A,B) and showed a marked age-related decrease in Prx III expression with a statistically significant 44%-reduced amount, shared by both AL-28 and AL-32 groups. The age-associated decrease in Prx III expression was attenuated in the CRO animals compared to the counterpart of the AL-MA group and such value was significantly higher than those of both AL-28 and AL-32 rats. Since a recent report [43] described the age-related accumulation of an oxidized sulphonic form of Prx III in rat hepatocytes, we searched, by western blotting experiments, the oxidized forms of various peroxiredoxins (PRX-SO_3_) in the four groups of animals (Figure 3 A,B). A relevant, statistically significant 33% increase of all PRX-SO_3_ forms was found in the AL-28 rats. In the AL-32 animals the amount of PRX-SO_3_ proteins was significantly reduced with respects to the AL-28 value and not statistically different from the AL-MA one. As for the CRO rats, their amount of oxidized peroxiredoxins was significantly lower than the AL-28 counterpart and not statistically different from that of the AL-MA group, supporting the preventive action of the CR towards the age-related oxidative damage of proteins. In relationship to the age-dependent oxidative stress, we determined the amount of the mitochondrial manganese-superoxide dismutase (SOD2), that is ROS- [44] and PGC-1α-induced [45], in all groups of rats (Figure 3 A,B). A consistent, marked age-related decrease was found in SOD2 expression with a statistically significant 24%-reduced amount in AL-28 rats and a 31%-decreased value in AL-32 animals. Such reduction was completely prevented by the CR since the CRO animals presented a value not statistically different with respects to that of the AL-MA group, but significantly higher than those of both AL-28 and AL-32 rats.

### Aging and CR effects on TFAM-binding at mtDNA origins of replication

**Figure 4:**
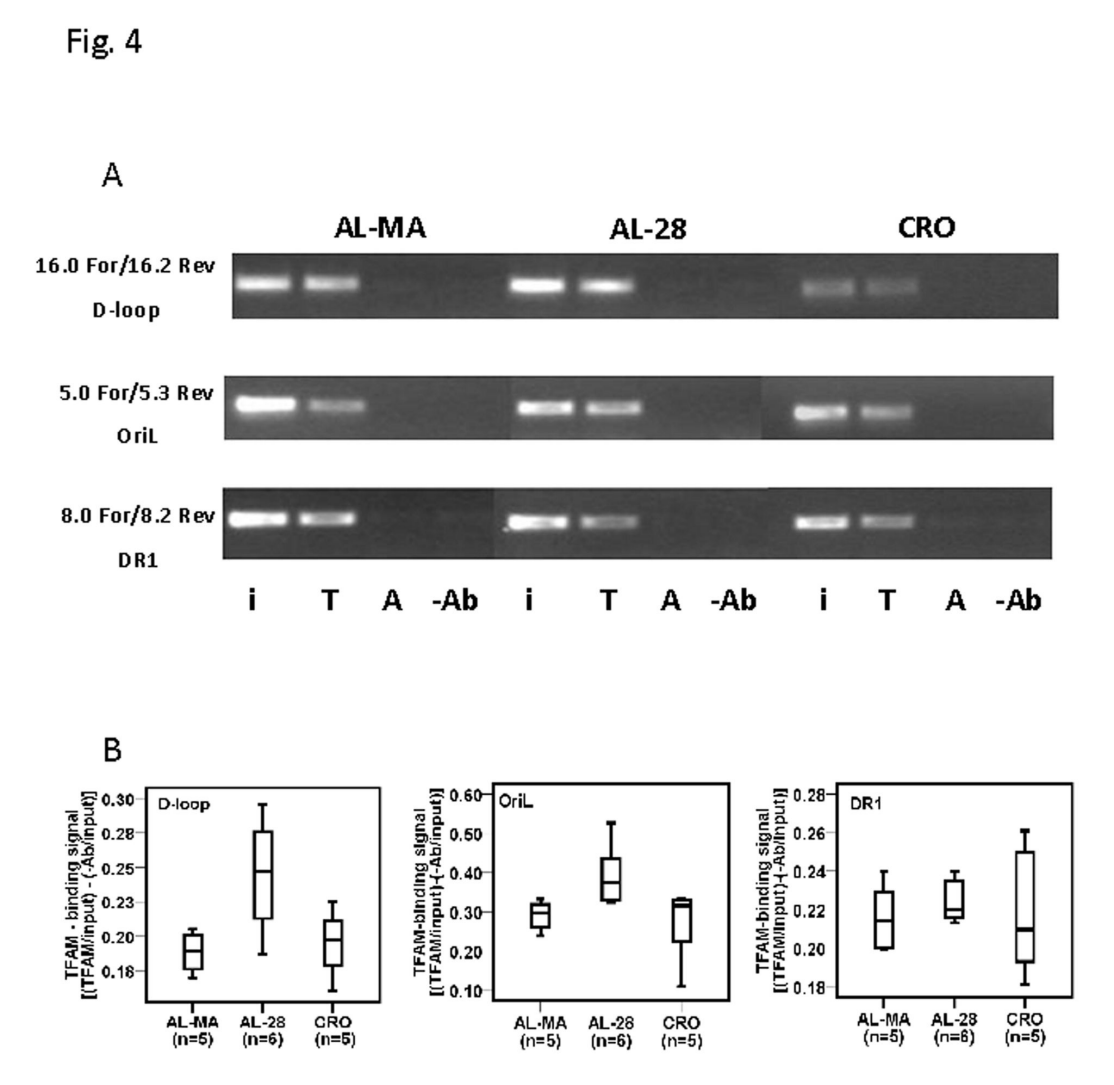
Age- and calorie restriction-related changes of TFAM binding to rat mtDNA regions by mIP assay. The semi-quantitative assay showed the presence of TFAM-binding at all assayed regions, while the densitometric evaluation of such results indicated the age-related increase in TFAM-binding at both D-loop and OriL regions, prevented by CR. **A** Representative results of semi-quantitative PCR amplifications of the mIP-derived templates from an AL-MA, an AL-28 and a CRO rat for the indicated regions. The specific primers pairs and the amplified genetic regions are indicated on the left side of the gel. The PCR reactions contained either input DNA (i) or mIP mtDNA immunoprecipitated with TFAM (T) or mIP mtDNA immunoprecipitated with β-actin (A) or mIP mtDNA immunoprecipitated with no antibody (-Ab). **B** Semi-quantitative evaluation by densitometry of TFAM binding in the three groups of animals after the mIP assay performed at three mtDNA regions. For each rat the signal value of every region was calculated in the three replicas by subtracting the value of the intensity of the aliquot precipitated without primary antibody from that of the TFAM-immunoprecipitated aliquot, both normalized to the value of the respective input aliquot made equal to 1. The respective mean was used in the box-plot representation of the corresponding group of rats. The “box” contains the middle 50% of the data (with the upper and the lower edges representing the 75^th^ and 25^th^ percentiles, respectively), the “horizontal line” within the box represents the median value. The filled lines indicate minimum and maximum data values for each rats group; n=number of analyzed animals.

**Figure 5:**
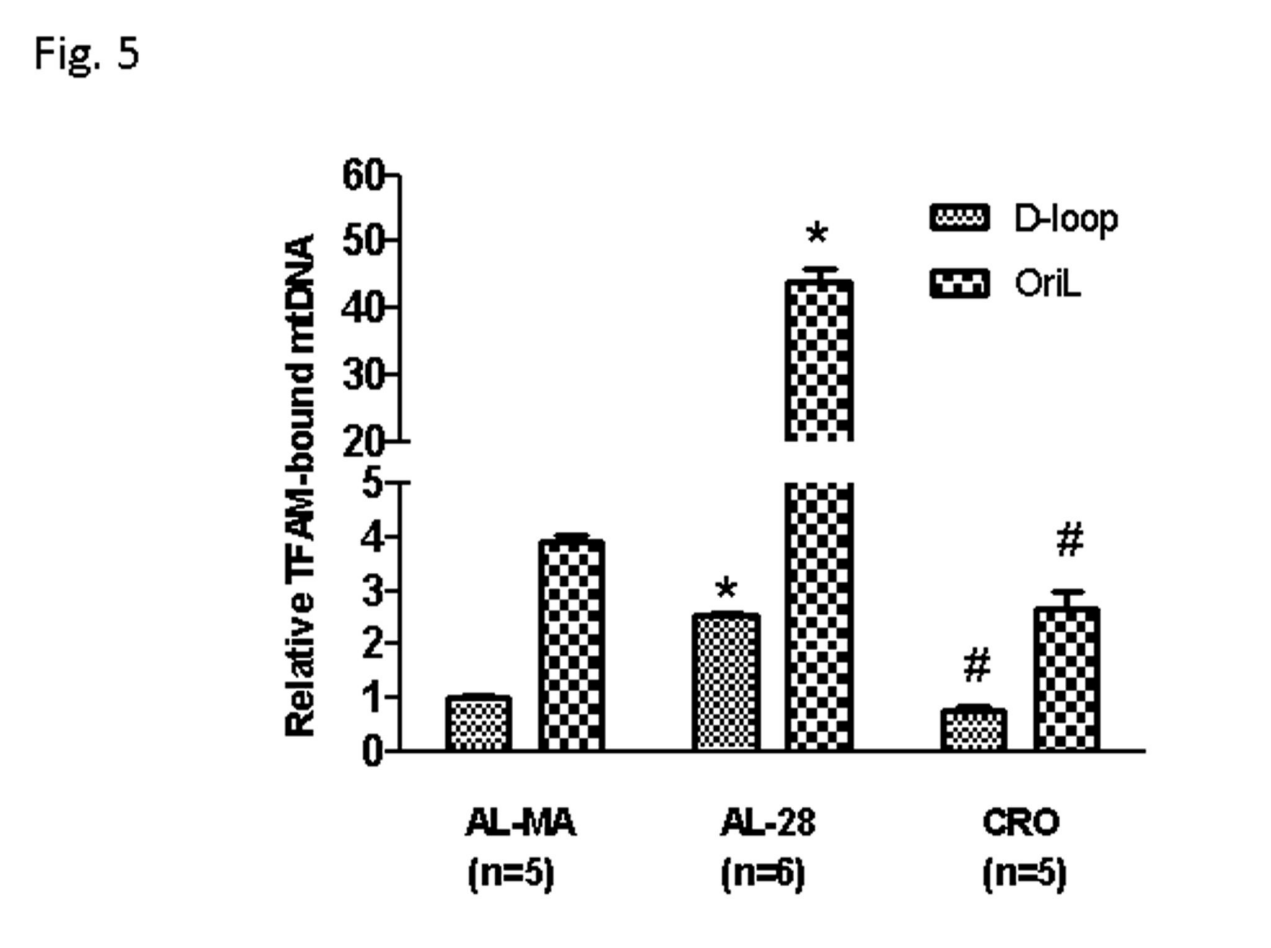
Age- and calorie restriction-related changes of TFAM-bound mtDNA amount at two mtDNA regions by RT-PCR. An age-related increase in the amount of TFAM-bound mtDNA was found at both assayed regions, while it was prevented by CR. The amplified products enclose sections, respectively, of the D-loop region and of the OriL region delimited by the primers pairs listed in the bottom part of Table 1. The calculation of the relative amount of TFAM-bound mtDNA was performed according to the formula 2^ΔCTx^ -2^ΔCTb^, where ΔCTx is the C_T_ difference between the C_T_ values of the input and of the immunoprecipitated sample and ΔCTb is the C_T_ difference between the C_T_ values of the input and of the –Ab sample. The obtained results were then normalized with respect to the mean value of TFAM-bound mtDNA in the D-loop region from AL-MA animals (fixed as 1). Bars represent the mean (+/- SD) of the relative amounts of TFAM-bound mtDNA derived from each of the assayed animals in the specific group. *p<0.05 versus the value of the AL-MA rats; # *p*<0.05 versus the amount from the same region of AL-28 rats; n=number of analyzed animals.

**Figure 6:**
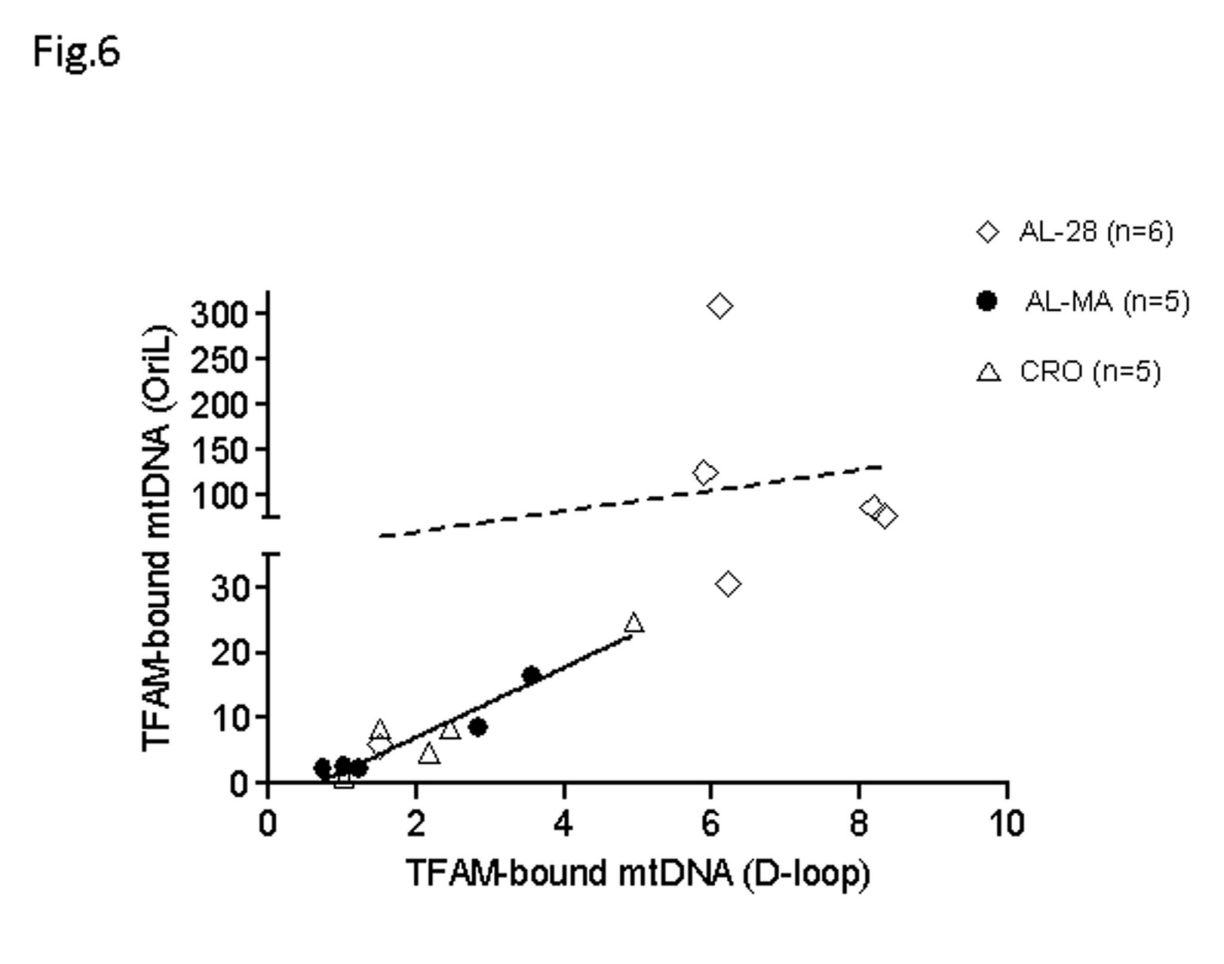
Correlation between TFAM-bound mtDNA amounts at the D-loop region and at the OriL region. A strong positive correlation was found between the TFAM-bound amounts of mtDNA at the assayed sub-regions in the joined animals from the AL-MA and CRO groups, but not in the AL-28 rats. The relative amounts of mtDNA immunoprecipitated by TFAM were determined by quantitative Real Time PCR (qRT-PCR) using primers for the D-loop and OriL sub-regions. Each sample was analyzed in triplicate. The calculation of the relative amount of TFAM-bound mtDNA was performed according to the formula 2^ΔCTx^ -2^ΔCTb^, where ΔCTx is the C_T_ difference between the C_T_ values of the input and of the immunoprecipitated sample and ΔCTb is the C_T_ difference between the C_T_ values of the input and of the –Ab sample. Pearson’s tests were performed to determine whether the amounts of TFAM-bound mtDNA at both origins of replication correlate with each other. The correlation was highly significant (p<0.005, correlation coefficient: 0.952) in the AL-MA and CRO joined groups (n=10, filled line); the correlation was not statistically significant and reached a lower value (p= N.S., correlation coefficient: 0.409) in the AL-28 group (n=6, dashed line).

Our recent study [13] demonstrated both age- and calorie restriction-related quantitative changes in the binding of TFAM to the two regions of mtDNA harbouring the origins of replication and indicated, respectively, as D-loop and OriL regions. Therefore, we decided to analyze the eventual presence and extent of changes in the *in vivo* mtDNA-binding specific activity of TFAM to three selected regions in rats from the AL-MA, AL-28 and CRO groups, by performing mtDNA immunoprecipitation (mIP) experiments followed by a semi-quantitative PCR assay. The assayed regions, encompassed, respectively: i) a part of the D-loop including the OriH origin of replication and the LSP promoter; ii) the OriL origin of replication with a portion of the COX I gene and iii) a sequence containing the Direct Repeat1 (DR1) of the 4.8-kb deletion. Such regions were chosen because of a possible different functional relevance between the D-loop and OriL regions, involved in mtDNA replication, and the DR1-cointaining region, likely representing all other mtDNA regions not involved in such process. In Figure 4 A from top to bottom are reported the representative results for the three regions. The semi-quantitative assay showed the presence of TFAM-binding in all assayed samples at the different regions. Figure 4 B reports the densitometric evaluation of the binding of TFAM at such regions in all tested rats in the box-plot form and indicates the age-related increase in TFAM-binding at both D-loop and OriL regions as specific for the AL-28 rats. It is also evident the absence of age- or diet-related changes in the binding of TFAM at the DR1-containing region. The analysis was deepened, using the same mIP-derived DNAs, by quantitative RT-PCR experiments performed in two sub-regions that derived from the original larger ones and enclosed, respectively, the promoter of the L-strand (LSP) from the D-loop region and a shorter sequence encompassing OriL and the surrounding stretch of tRNA genes. The quantitative results are presented in Figure 5 and demonstrated an age-related, statistically significant increase in the amount of TFAM-bound mtDNA at both assayed regions in the AL-28 rats. The age-matched CRO animals, on the contrary, did not show any statistically significant difference with respect to the younger AL-MA group at the two regions, supporting a preventive action by the CR, likely able to maintain, in spite of aging, the necessary TFAM-binding activity. Since the mean value of the TFAM-bound mtDNA amount at the OriL sub-region appeared generally higher than its D-loop counterpart, we decided to analyze the corresponding paired values from all samples. It was thus found a statistically very significant positive correlation (p= 0.0001, correlation coefficient: 0.952) between the TFAM-bound amounts of mtDNA at the assayed sub-regions in all animals from the joined AL-MA and CRO groups (Figure 6, filled line). Such direct correlation was not statistically significant and reached a lower value coefficient (p= N.S., correlation coefficient: 0.409), in the AL-28 animals (Figure 6, dashed line) suggesting a different trend with respect to the AL-MA and CRO counterparts.

## Discussion

### Aging and CR effects on mitochondrial biogenesis

Rat liver demonstrates with aging a marked mitochondrial dysfunction [4] and an accumulation of oxidative damages to mitochondrial proteins [43], lipids [46] and DNA [32,34,35]. In particular, rat liver mtDNA presents also an age-related decrease in its relative content [10,14,15], accompanied by a concordant decrease in TFAM amount [15], thus raising a number of questions about the effects of aging on organelle biogenesis. Long-term CR has been shown to be very effective in liver [34], completely preventing the age-related loss of mtDNA [14]. The samples in the present study derived from the very long-living hybrid rat strain Fischer 344 x Brown Norway, allowing the use of the animals included in the middle age group (AL-MA) as controls, being at the beginning of the aging process, for comparisons with the old (AL-28), the very old (AL-32) and the old calorie-restricted (CRO) groups. The determination of mtDNA content and TFAM amount in the three AL-fed groups demonstrated an age-related decrease, more marked for TFAM than for the mtDNA. The age-reduced amount of TFAM might be related to the loss of mtDNA in the AL-fed animals if it induced something similar to what has been recently reported in TFAM heterozygous knockout mice, presenting decreased TFAM and mtDNA content together with increased oxidative mtDNA damage [47]. Considering the histon-like function of TFAM and its possible involvement in the repair mechanisms of mtDNA [24], its age-reduced amount might decrease both the protection against the mtDNA oxidative damage [48] and the performance of the repairing enzymes. MtDNA content and TFAM age-related decreases were completely prevented by CR, supporting a clear preventive action of the CR during the lifespan of the animals. We then examined the expression of two major factors active in the mitochondrial biogenesis cascade leading to TFAM namely PGC-1α and NRF-1 as well as of a nuclear DNA-encoded subunit of the cytochrome c oxidase complex that is COX IV, also regulated by PGC-1α [40] and NRF-1 [41], and usually expressed in a coordinated way with the mtDNA-encoded subunits. The age-related decreased expression was shared by all three assayed proteins in the AL-28 rats and the reduction was maintained or even further enhanced in the AL-32 animals. This indicates a marked decrease in the whole mitochondrial biogenesis process with aging and is in good agreement with the results obtained in liver from aged Fisher 344 rats [15]. We could also demonstrate in the present work the ability of the CR to fully preserve the mitochondrial biogenesis pathway in rat liver, allowing the maintenance of MA-like values in spite of aging. Since previous reports described the age-related oxidative stress of liver [34], we analyzed the amounts of some proteins usually influenced by oxidative stress namely the well-known mitochondrial H_2_O_2_ scavenging protein Prx III [42], the oxidized peroxiredoxins [43] and the ROS-induced, PGC-1α-regulated SOD2 enzyme [44,45]. Effectively an increased presence of ROS was consistent with the raised amount of oxidized peroxiredoxins found in the AL-28 rats. The decreased amount of oxidized peroxiredoxins in the AL-32 animals with respects to the highest value in the AL-28 animals might be explained supposing that the very small number of rats reaching the oldest age included the animals most able to cope with the age-related oxidative stress, whereas the more numerous rats not surviving until 32 months of age comprised those less able to face such condition. This result is also in good agreement with the finding in the old group of the less long-living Fisher 344 rats reported by Pesce et al. [15]. CR was able to reduce the accumulation of oxidized peroxiredoxins with respect to the value of the age-matched AL counterpart, supporting the preventive action of the CR against the age-related oxidative stress. In spite of the verified age-related oxidative damage to proteins we found a reduced amount of the ROS-scavenging protein Prx III in AL-28 and AL-32 rats. These unexpected results could be explained if the age-related reduction of mitochondrial biogenesis, also including Prx III expression [49], were the final outcome, prevailing above the expected ROS-induced up-regulation of Prx III expression [50,51]. CR was able to partially prevent the age-related down-regulation of Prx III expression probably through the induction of mitochondrial biogenesis, supporting the preventive effect of the CR. Finally, the SOD2 amounts were consistent with the reduced PGC-1α expression, indicating in AL-28 and AL-32 rats the age-related decrease in mitochondrial biogenesis as the prevailing effect above the oxidative stress trigger, expected also for SOD2 regulation [44]. Furthermore, the age-related decrease in SOD2 protein amounts is in good agreement with the SOD2 age-decreased value reported by Pesce et al. [15]. CR with its induction of mitochondrial biogenesis, vice versa, maintained a condition for SOD2 expression very similar to that of the AL-MA rats, delaying the aging effect.

### Aging and CR effects on TFAM binding to mtDNA origins of replication

In our recent study we analyzed the changes in mtDNA content, TFAM amount and TFAM binding to mtDNA in frontal cortex from aged rats fed *ad libitum* or with CR. It was thus possible to unveil a novel, brain-specific mechanism, implying changes in TFAM binding to some regions of mtDNA and likely responsible for the reported changes in TFAM and mtDNA contents [13]. In the present study we examined, by quantitative RT-PCR, the amounts of TFAM-bound mtDNA at the two sub-regions involved in mtDNA replication. It was found in the AL-28 rats an age-related increase in the mtDNA amounts bound by TFAM at both sub-regions encompassing, respectively, LSP and OriL, whereas CR preserved in the old rats amounts of TFAM-bound mtDNA not different from those of the AL-MA counterparts. The age-related increase in TFAM binding at both D-loop and OriL sub-regions was unexpected, especially considering the mtDNA loss occurring in the same animals. In addition to that, the mean mtDNA amounts bound by TFAM at the D-loop and the OriL sub-regions appeared coordinated between themselves in all examined groups, suggesting some kind of functional relationship. Effectively, by Pearson’s test analysis, we identified a completely novel, highly significant direct correlation between the paired amounts of TFAM-bound mtDNA at these sub-regions in the joined AL-MA and CRO groups. Such finding reinforced the possibility of a coordinated modulation of TFAM binding to the two sub-regions because of their functional role in mtDNA replication. Furthermore, the large similarity between the AL-MA and the CRO groups strongly indicated that CR was able to preserve in the old animals those parameters modulating TFAM binding in a MA-like condition. This was another clue supportive of the effect implied at mitochondrial molecular level in the aging process by the CR. On the contrary, aging led to increased amounts of TFAM-bound mtDNA at both sub-regions. These data, on overall, supported a coordinated, functionally-related modulation of TFAM binding at the two regions including LSP and OriL which appears involved in the differential regulation of mitochondrial biogenesis elicited by aging either CR. We could also infer from the respective samples results (data not shown) that in the AL-28 rats, featuring mtDNA contents smaller than those of the AL-MA+CRO counterparts in spite of the larger amounts of TFAM-bound mtDNA, the increased TFAM binding at both origins somehow prevented an efficient mtDNA replication process, leading to the corresponding loss of mtDNA. In particular, the amounts of TFAM-bound mtDNA at the OriL sub-region were always higher than the corresponding ones at the D-loop sub-region, but such difference became very relevant in five out of the six AL-28 rats plotted in Figure 6 and we hypothesize that such massive binding of TFAM at OriL might negatively affect the mtDNA replication. It might occur with aging something similar to what has been described for TFAM binding at the HSP2 promoter region in the regulation of transcription examined by *in vitro* titration experiments, where it has been demonstrated that increasing TFAM concentrations above a certain threshold prevented rather than enhanced the transcription process [52-55]. Furthermore, it was reported the absence of an age-related decrease in mitochondrial transcription in rat liver [56,10], eventually consistent with a threshold, not exceeded by the slightly increased TFAM-binding at the D-loop sub-region. Vice versa, the CRO animals presented amounts of TFAM-bound mtDNA at both origins of replication smaller than those of the AL-28 rats and yet they were characterized by specific mtDNA contents (data not shown) even larger than those of the AL-MA animals. This was highly suggestive of the full preservation of TFAM functions with CR in spite of aging and it might be related to the general reprogramming of the mitochondrial protein acetylome, including TFAM, described in calorie-restricted mice [57], that might affect also the mtDNA-binding activity of the factor. Considering all together the present results, we demonstrate in rat liver a very articulated age-related decrease in mitochondrial biogenesis leading to the loss of mtDNA probably also through the increase of TFAM binding to both origins of replication. The reduction of mitochondrial biogenesis appears prevailing above the oxidative stress condition in the final outcome of the age-related regulation of expression of some mitochondrial proteins. This gives an interesting and novel clue to evaluate the preservation of mitochondrial biogenesis as very relevant in the anti-aging action of CR. Of course, future work will be necessary to further verify such hypothesis also in consideration of the therapeutic applications that might lead, through up-regulation of PGC-1α expression and maintenance of mtDNA, to a longer-lasting mitochondrial functionality.

## References

[1] Wallace DC (1992) Mitochondrial genetics: a paradigm for aging and degenerative diseases? Science 256: 628-632. doi:10.1126/science.1533953. PubMed: 1533953. 1533953 10.1126/science.1533953

[2] Huang JH, Hood DA (2009) Age-associated mitochondrial dysfunction in skeletal muscle: Contributing factors and suggestions for long-term interventions. IUBMB Life 61: 201-214. doi:10.1002/iub.164. PubMed: 19243006. 19243006 10.1002/iub.164

[3] Navarro A, Boveris A (2010) Brain mitochondrial dysfunction in aging, neurodegeneration, and Parkinson’s disease. Front Aging Neurosci 2: 1-11. PubMed: 20552041. 20890446 10.3389/fnagi.2010.00034 PMC2947925

[4] Navarro A, Boveris A (2007) The mitochondrial energy transduction system and the aging process. Am J Physiol Cell Physiol 292: C670-C686. PubMed: 17020935. 17020935 10.1152/ajpcell.00213.2006

[5] Harman D (1972) The biologic clock: the mitochondria? J Am Geriatr Soc 20: 145-147. PubMed: 5016631. 5016631 10.1111/j.1532-5415.1972.tb00787.x

[6] Miguel J, Fleming JE (1986) Theoretical and experimental support for an “oxygen radical mitochondrial injury” hypothesis of cell aging. In: Johnson J Walford R Harman D Miquel J Free Radicals, Aging, and Degenerative Diseases. ALAN Rev Liss: 51–74.

[7] Linnane AW, Marzuki S, Ozawa T, Tanaka M (1989) Mitochondrial DNA mutations as an important contributor to ageing and degenerative diseases. Lancet 1: 642-645. PubMed: 2564461. 2564461 10.1016/s0140-6736(89)92145-4

[8] Druzhyna NM, Wilson GL, LeDoux SP (2008) Mitochondrial DNA repair in aging and disease. Mech Ageing Dev 129: 383-390. doi:10.1016/j.mad.2008.03.002. PubMed: 18417187. 18417187 10.1016/j.mad.2008.03.002 PMC2666190

[9] Barrientos A, Casademont J, Cardellach F, Estivill X, Urbano-Marquez A et al. (1997) Reduced steady-state levels of mitochondrial RNA and increased mitochondrial DNA amount in human brain with aging. Brain Res. Mol Brain Res 52: 284-289. doi:10.1016/S0169-328X(97)00278-7. PubMed: 9495550. 9495550 10.1016/s0169-328x(97)00278-7

[10] Barazzoni R, Short KR, Nair KS (2000) Effects of aging on mitochondrial DNA copy number and cytochrome c oxidase gene expression in rat skeletal muscle, liver, and heart. J Biol Chem 275: 3343-3347. doi:10.1074/jbc.275.5.3343. PubMed: 10652323. 10652323 10.1074/jbc.275.5.3343

[11] Pesce V, Cormio A, Fracasso F, Vecchiet J, Felzani G et al. (2001) Age-related mitochondrial genotypic and phenotypic alterations in human skeletal muscle. Free Radic Biol Med 30: 1223-1233. doi:10.1016/S0891-5849(01)00517-2. PubMed: 11368920. 11368920 10.1016/s0891-5849(01)00517-2

[12] McInerny SC, Brown AL, Smith DW (2009) Region-specific changes in mitochondrial D-loop in aged rat CNS. Mech Ageing Dev 130: 343-349. doi:10.1016/j.mad.2009.01.008. PubMed: 19428453. 19428453 10.1016/j.mad.2009.01.008

[13] Picca A, Fracasso F, Pesce V, Cantatore P, Joseph AM et al. Age- and calorie restriction-related changes in rat brain mitochondrial DNA and TFAM binding. Dordr: AGE . doi:10.1007/s11357-012-9465-z Sep 4 (2012) [Epub ahead of print]. 10.1007/s11357-012-9465-z PMC3776104 22945739

[14] Cassano P, Sciancalepore AG, Lezza AM, Leeuwenburgh C, Cantatore P et al. (2006) Tissue-specific effect of age and caloric restriction diet on mitochondrial DNA content. Rejuvenation Res 9: 211-214. doi:10.1089/rej.2006.9.211. PubMed: 16706645. 16706645 10.1089/rej.2006.9.211

[15] Pesce V, Nicassio L, Fracasso F, Musicco C, Cantatore P et al. (2012) Acetyl-L-carnitine activates the peroxisome proliferator-activated receptor-γ coactivators PGC-1α/ PGC-1β-dependent signaling cascade of mitochondrial biogenesis and decreases the oxidized peroxiredoxins content in old rat liver. Rejuvenation Res 15: 136-139. doi:10.1089/rej.2011.1255. PubMed: 22533417. 22533417 10.1089/rej.2011.1255

[16] Sahin E, DePinho RA (2012) Axis of ageing: telomeres, p53 and mitochondria. Nat Rev Mol Cell Biol 13: 397-404. doi:10.1038/nrm3352. PubMed: 22588366. 22588366 10.1038/nrm3352 PMC3718675

[17] Kanki T, Ohgaki K, Gaspari M, Gustafsson CM, Fukuoh A et al. (2004) Architectural role of mitochondrial transcription factor A in maintenance of human mitochondrial DNA. Mol Cell Biol 24: 9823-9834. doi:10.1128/MCB.24.22.9823-9834.2004. PubMed: 15509786. 15509786 10.1128/MCB.24.22.9823-9834.2004 PMC525493

[18] Lezza AMS (2012) Mitochondrial transcription factor A (TFAM): one actor for different roles. Front Biol 7: 30–39. doi:10.1007/s11515-011-1175-x.

[19] Fisher RP, Clayton DA (1988) Purification and characterization of human mitochondrial transcription factor 1. Mol Cell Biol 8: 3496-3509. PubMed: 3211148. 3211148 10.1128/mcb.8.8.3496-3509.1988 PMC363587

[20] Larsson NG, Wang J, Wilhelmsson H, Oldfors A, Rustin P et al. (1998) Mitochondrial transcription factor A is necessary for mtDNA maintenance and embryogenesis in mice. Nat Genet 18: 231-236. doi:10.1038/ng0398-231. PubMed: 9500544. 9500544 10.1038/ng0398-231

[21] Parisi MA, Clayton DA (1991) Similarity of human mitochondrial transcription factor 1 to high mobility group proteins. Science 252: 965-969. doi:10.1126/science.2035027. PubMed: 2035027. 2035027 10.1126/science.2035027

[22] Kaufman BA, Durisic N, Mativetsky JM, Costantino S, Hancock MA et al. (2007) The mitochondrial transcription factor TFAM coordinates the assembly of multiple DNA molecules into nucleoid-like structures. Mol Biol Cell 18: 3225-3236. doi:10.1091/mbc.E07-05-0404. PubMed: 17581862. 17581862 10.1091/mbc.E07-05-0404 PMC1951767

[23] Yoshida Y, Izumi H, Ise T, Uramoto H, Torigoe T et al. (2002) Human mitochondrial transcription factor A binds preferentially to oxidatively damaged DNA. Biochem Biophys Res Commun 295: 945-951. doi:10.1016/S0006-291X(02)00757-X. PubMed: 12127986. 12127986 10.1016/s0006-291x(02)00757-x

[24] Canugovi C, Maynard S, Bayne AC, Sykora P, Tian J et al. (2010) The mitochondrial transcription factor A functions in mitochondrial base excision repair. DNA Repair 9: 1080-1089. doi:10.1016/j.dnarep.2010.07.009. PubMed: 20739229. 20739229 10.1016/j.dnarep.2010.07.009 PMC2955416

[25] Dinardo MM, Musicco C, Milella F, Gadaleta MN et al. (2003) Acetylation and level of mitochondrial transcription factor A in several organs of young and old rats. Biochem Biophys Res Commun 301: 187-191. doi:10.1016/S0006-291X(02)03008-5. PubMed: 12535660. 12535660 10.1016/s0006-291x(02)03008-5

[26] Pesce V, Cormio A, Fracasso F, Lezza AM, Cantatore P et al. (2005) Age-related changes of mitochondrial DNA content and mitochondrial genotypic and phenotypic alterations in rat hind-limb skeletal muscles. J Gerontol A Biol Sci Med Sci 60: 715-723. doi:10.1093/gerona/60.6.715. PubMed: 15983173. 15983173 10.1093/gerona/60.6.715

[27] Masoro EJ (2000) Caloric restriction and aging: an update. Exp Gerontol 35: 299-305. doi:10.1016/S0531-5565(00)00084-X. PubMed: 10832051. 10832051 10.1016/s0531-5565(00)00084-x

[28] Civitarese AE, Carling S, Heilbronn LK, Hulver MH, Ukropcova B et al. (2007) Calorie restriction increases muscle mitochondrial biogenesis in healthy humans. PLOS Med 4: e76. doi:10.1371/journal.pmed.0040076. PubMed: 17341128. 17341128 10.1371/journal.pmed.0040076 PMC1808482

[29] Guarente L (2008) Mitochondria-a nexus for aging, calorie restriction, and sirtuins? Cell 132: 171-176. doi:10.1016/j.cell.2008.01.007. PubMed: 18243090. 18243090 10.1016/j.cell.2008.01.007 PMC2680180

[30] Barja G (2007) Mitochondrial oxygen consumption and reactive oxygen species production are independently modulated: implications for aging studies. Rejuvenation Res 10: 215-224. doi:10.1089/rej.2006.0516. PubMed: 17523876. 17523876 10.1089/rej.2006.0516

[31] Aspnes LE, Lee CM, Weindruch R, Chung SS, Roecker EB et al. (1997) Caloric restriction reduces fiber loss and mitochondrial abnormalities in aged rat muscle. FASEB J 11: 573-581. PubMed: 9212081. 9212081 10.1096/fasebj.11.7.9212081

[32] Kang CM, Kristal BS, Yu BP (1998) Age-related mitochondrial DNA deletions: effect of dietary restriction. Free Radic Biol Med 24: 148-154. doi:10.1016/S0891-5849(97)00204-9. PubMed: 9436624. 9436624 10.1016/s0891-5849(97)00204-9

[33] Gredilla R, Barja G, López-Torres M (2001) Effect of short-term caloric restriction on H_2_O_2_ production and oxidative DNA damage in rat liver mitochondria and location of the free radical source. J Bioenerg Biomembr 33: 279-287. doi:10.1023/A:1010603206190. PubMed: 11710804. 11710804 10.1023/a:1010603206190

[34] López-Torres M, Gredilla R, Sanz A, Barja G (2002) Influence of aging and long-term caloric restriction on oxygen radical generation and oxidative DNA damage in rat liver mitochondria. Free Radic Biol Med 32: 882-889. doi:10.1016/S0891-5849(02)00773-6. PubMed: 11978489. 11978489 10.1016/s0891-5849(02)00773-6

[35] Cassano P, Lezza AM, Leeuwenburgh C, Cantatore P, Gadaleta MN (2004) Measurement of the 4,834-bp mitochondrial DNA deletion level in aging rat liver and brain subjected or not to caloric restriction diet. Ann N Y Acad Sci 1019: 269-273. doi:10.1196/annals.1297.045. PubMed: 15247027. 15247027 10.1196/annals.1297.045

[36] Stuart JA, Karahalil B, Hogue BA, Souza-Pinto NC, Bohr VA (2004) Mitochondrial and nuclear DNA base excision repair are affected differently by caloric restriction. FASEB J 18: 595-597. PubMed: 14734635. 14734635 10.1096/fj.03-0890fje

[37] Bua E, McKiernan SH, Aiken JM (2004) Calorie restriction limits the generation but not the progression of mitochondrial abnormalities in aging skeletal muscle. FASEB J 18: 582-584. PubMed: 14734641. 14734641 10.1096/fj.03-0668fje

[38] Pfaffl MW (2001) A new mathematical model for relative quantification in real-time PCR. Nucleic Acids Res 29: e45. doi:10.1093/nar/29.9.e45. PubMed: 11328886. 11328886 10.1093/nar/29.9.e45 PMC55695

[39] Vercauteren K, Pasko RA, Gleyzer N, Marino VM, Scarpulla RC (2006) PGC-1-related coactivator: immediate early expression and characterization of a CREB/NRF-1 binding domain associated with cytochrome c promoter occupancy and respiratory growth. Mol Cell Biol 26: 7409-7419. doi:10.1128/MCB.00585-06. PubMed: 16908542. 16908542 10.1128/MCB.00585-06 PMC1636882

[40] Anderson RM, Barger JL, Edwards MG, Braun KH, O’Connor CE et al. (2008) Dynamic regulation of PGC-1α localization and turnover implicates mitochondrial adaptation in calorie restriction and the stress response. Aging Cell 7: 101-111. doi:10.1111/j.1474-9726.2007.00357.x. PubMed: 18031569. 18031569 10.1111/j.1474-9726.2007.00357.x PMC2253697

[41] Scarpulla RC (2002) Nuclear activators and coactivators in mammalian mitochondrial biogenesis. Biochim Biophys Acta 1576: 1-14. doi:10.1016/S0167-4781(02)00343-3. PubMed: 12031478. 12031478 10.1016/s0167-4781(02)00343-3

[42] Chang TS, Cho CS, Park S, Yu S, Kang SW, Rhee SG (2004) Peroxiredoxin III, a mitochondrion-specific peroxidase, regulates apoptotic signaling by mitochondria. J Biol Chem 279: 41975-41984. doi:10.1074/jbc.M407707200. PubMed: 15280382. 15280382 10.1074/jbc.M407707200

[43] Musicco C, Capelli C, Pesce V, Timperio AM, Calvani M et al. (2009) Accumulation of overoxidized Peroxiredoxin III in aged rat liver mitochondria. Biochim Biophys Acta 1787: 890-896. doi:10.1016/j.bbabio.2009.03.002. PubMed: 19272351. 19272351 10.1016/j.bbabio.2009.03.002

[44] Storz P, Döppler H, Toker A (2005) Protein kinase D mediates mitochondrion-to-nucleus signaling and detoxification from mitochondrial reactive oxygen species. Mol Cell Biol 25: 8520-8530. doi:10.1128/MCB.25.19.8520-8530.2005. PubMed: 16166634. 16166634 10.1128/MCB.25.19.8520-8530.2005 PMC1265746

[45] St-Pierre J, Drori S, Uldry M, Silvaggi JM, Rhee J et al. (2006) Suppression of reactive oxygen species and neurodegeneration by the PGC-1α transcriptional coactivators. Cell 127: 397-408. doi:10.1016/j.cell.2006.09.024. PubMed: 17055439. 17055439 10.1016/j.cell.2006.09.024

[46] Paradies G, Petrosillo G, Paradies V, Ruggiero FM (2010) Oxidative stress, mitochondrial bioenergetics, and cardiolipin in aging. Free Radic Biol Med 48: 1286-1295. doi:10.1016/j.freeradbiomed.2010.02.020. PubMed: 20176101. 20176101 10.1016/j.freeradbiomed.2010.02.020

[47] Woo DK, Green PD, Santos JH, D’Souza AD, Walther Z, Martin WD, Christian BE, Chandel NS, Shadel GS (2012) Mitochondrial genome instability and ROS enhance intestinal tumorigenesis in APC (Min/+) mice. Am J Pathol 180: 24-31. doi:10.1016/j.ajpath.2011.10.003. PubMed: 22056359. 22056359 10.1016/j.ajpath.2011.10.003 PMC3338350

[48] Van Houten B, Woshner V, Santos JH (2006) Role of mitochondrial DNA in toxic responses to oxidative stress. DNA Repair (Amst) 5: 145-152. doi:10.1016/j.dnarep.2005.03.002. PubMed: 15878696. 15878696 10.1016/j.dnarep.2005.03.002

[49] Huh JY, Kim Y, Jeong J, Park J, Kim I et al. (2012) Peroxiredoxin 3 is a key molecule regulating adipocyte oxidative stress, mitochondrial biogenesis, and adipokine expression. Antioxid Redox Signal 16: 229-243. doi:10.1089/ars.2010.3766. PubMed: 21902452. 21902452 10.1089/ars.2010.3766 PMC3234662

[50] Chiribau CB, Cheng L, Cucoranu IC, Yu YS, Clempus RE et al. (2008) FOXO3A regulates peroxiredoxin III expression in human cardiac fibroblasts. J Biol Chem 283: 8211-8217. doi:10.1074/jbc.M710610200. PubMed: 18195003. 18195003 10.1074/jbc.M710610200 PMC2276380

[51] Hwang IK, Yoo KY, Kim DW, Lee CH, Choi JH et al. (2010) Changes in the expression of mitochondrial peroxiredoxin and thioredoxin in neurons and glia and their protective effects in experimental cerebral ischemic damage. Free Radic Biol Med 48: 1242-1251. doi:10.1016/j.freeradbiomed.2010.02.007. PubMed: 20156553. 20156553 10.1016/j.freeradbiomed.2010.02.007

[52] Dairaghi DJ, Shadel GS, Clayton DA (1995) Human mitochondrial transcription factor A and promoter spacing integrity are required for transcription initiation. Biochim Biophys Acta 1271: 127-134. doi:10.1016/0925-4439(95)00019-Z. PubMed: 7599198. 7599198 10.1016/0925-4439(95)00019-z

[53] Lodeiro MF, Uchida A, Bestwick M, Moustafa IM, Arnold JJ et al. (2012) Transcription from the second heavy-strand promoter of human mtDNA is repressed by transcription factor A in vitro. Proc Natl Acad Sci U S A 109: 6513-6518. doi:10.1073/pnas.1118710109. PubMed: 22493245. 22493245 10.1073/pnas.1118710109 PMC3340051

[54] Zollo O, Tiranti V, Sondheimer N (2012) Transcriptional requirements of the distal heavy-strand promoter of mtDNA. Proc Natl Acad Sci U S A 109: 6508-6512. doi:10.1073/pnas.1118594109. PubMed: 22454497. 22454497 10.1073/pnas.1118594109 PMC3340101

[55] Shutt TE, Bestwick M, Shadel GS (2011) The core human mitochondrial transcription initiation complex. It only takes two to tango. Transcription 2: 55–59. doi:10.4161/trns.2.2.14296. PubMed: 21468229. 21468229 10.4161/trns.2.2.14296 PMC3062394

[56] Gadaleta MN, Petruzzella V, Renis M, Fracasso F, Cantatore P (1990) Reduced transcription of mitochondrial DNA in the senescent rat. Tissue dependence and effect of L-carnitine. Eur J Biochem 187: 501–506. doi:10.1111/j.1432-1033.1990.tb15331.x. PubMed: 2154375. 2154375 10.1111/j.1432-1033.1990.tb15331.x

[57] Hebert AS, Dittenhafer-Reed KE, Yu W, Bailey DJ, Selen ES et al. (2013) Calorie restriction and SIRT3 trigger global reprogramming of the mitochondrial protein acetylome. Mol Cell 49: 186-199. PubMed: 23201123. 23201123 10.1016/j.molcel.2012.10.024 PMC3704155

